# Evolution of Physiological Responses and Fatigue Analysis in Padel Matches According to Match Outcome and Playing Position

**DOI:** 10.3390/s25175240

**Published:** 2025-08-23

**Authors:** Bingen Marcos-Rivero, Javier Yanci, Cristina Granados, Jon Mikel Picabea, Josu Ascondo

**Affiliations:** 1 Research Group in Physical Activity, Exercise, and Sport (AKTIBOki), Department of Physical and Sports Education, Faculty of Education and Sport, University of the Basque Country (UPV/EHU), 01007 Vitoria-Gasteiz, Spain; bingen.marcos@ehu.eus (B.M.-R.); cristina.granados@ehu.eus (C.G.); josu.ascondo@ehu.eus (J.A.); 2 Society, Sports and Physical Exercise Research Group (GIKAFIT), Physical Education and Sport Department, Faculty of Education and Sport, University of the Basque Country (UPV/EHU), 01007 Vitoria-Gasteiz, Spain; jonmikel.picabea@ehu.eus

**Keywords:** match demands, heart rate, recovery, sport, workload

## Abstract

Padel is a doubles racket sport played on an enclosed court, characterised by intermittent high-intensity efforts, frequent directional changes, and short recovery periods. This study aimed to analyse the evolution of physiological responses and neuromuscular fatigue in amateur padel players according to playing position (Right Side [RS] vs. Left Side [LS]) and match outcome (Win or Lose). A total of 52 padel players (35.6 ± 11.6 years) participated, competing in 13 matches. The mean match duration was 57.2 ± 15.7 min, with an average of 152.0 ± 40.4 points per match. Physiological variables were recorded during each set, and neuromuscular variables (countermovement jump [CMJ] and handgrip strength) were assessed before the match and after each set. No significant differences in physiological load were found between winners and losers or between RS and LS positions. However, differences in handgrip strength were observed at T1 (*p* < 0.05, d = −0.72) and T2 (*p* < 0.05, d = −0.59) (post-set testing), with LS players showing higher grip strength. Regarding the progression of physiological responses across the different sets, a progressive increase in cardiovascular load was observed within each subgroup, with significant differences across sets (set 1, set 2, and set 3) in several variables, including HR_peak_, HR_avg_, zone 1, zone 2, zone 3, and TRIMP_Edwards_. No performance decline was observed in CMJ or handgrip strength in any of the groups analysed. These findings suggest that physiological responses increase throughout a match, particularly in the final sets, but no signs of neuromuscular fatigue (CMJ and handgrip) were observed, regardless of match outcome or playing position. These results highlight the need to include high-intensity scenarios and role-specific strategies in training to address the progressive physiological demands and positional differences in match play.

## 1. Introduction

Padel is a complex sport in which performance is determined by the interaction of multiple factors, including physiological and physical capacities, psychological skills, and technical and tactical abilities [1]. This sport is played in pairs on an enclosed court measuring 20 m in length by 10 m in width, bounded by glass walls and metal mesh, both of which are active components of the game [2]. In recent decades, padel has experienced exponential growth worldwide [3], with millions of players actively participating in numerous countries [4].

Current scientific knowledge on padel performance primarily focuses on the physical demands of the game, including specific variables such as rally duration, number of strokes per point, and effective playing time, as well as the effectiveness of specific technical actions across different areas of the court [5]. A recent study involving 83 professional padel players and analysing 23 matches found that winning players covered significantly greater distances and performed a higher number of accelerations per hour of play compared to losing players [6]. Furthermore, Miralles et al. [7] observed that professional players positioned on the Left Side exhibited a higher frequency of accelerations and decelerations than those playing on the Right Side. These recent studies suggest that both match outcome (Win or Loss) and court position (Right Side or Left Side) may be contextual variables influencing the physical responses of professional padel players.

From a physical and physiological demand perspective, playing a padel match involves intermittent efforts, characterised by a high number of strokes and repeated high-intensity actions such as accelerations, decelerations, and changes in direction [1]. On average, players cover approximately 3775 m, perform over 600 accelerations, accumulate around 63 AU of player load, and complete nearly 420 m of explosive distance, highlighting the sport’s demanding neuromuscular and locomotor profile [6]. Additionally, players reported average heart rate values of 143.63 ± 12.17 bpm, further evidencing the substantial cardiovascular demands of padel [8]. Given the demanding nature of these intermittent, high-intensity efforts, which may lead to substantial fatigue in athletes, it is of particular interest to quantify the fatigue potentially induced by competition in this sport. Fatigue is a complex phenomenon that affects both physical and mental performance, manifesting as a reduction in neuromuscular capacity to generate force and as mental fatigue, leading to tiredness and impaired cognitive performance following prolonged exertion [9,10]. Fatigue was assessed using different instruments that allow for the evaluation of both neuromuscular and functional dimensions. Specifically, CMJ and handgrip strength were used as key indicators [11]. These tests have been widely used in the scientific literature to evaluate the onset of fatigue, as they can detect potential alterations in muscular performance throughout repeated or prolonged efforts, such as those occurring during a padel match [12].

While fatigue induced by competition has been widely investigated in other sports disciplines [13,14], few studies have specifically analysed competition-induced fatigue in padel [15], and to date, no research has examined neuromuscular fatigue or the evolution of physiological responses in amateur padel matches. Moreover, it remains unclear whether such fatigue may be influenced by factors such as playing position, match outcome, or variables including playing time and the number of points played. In this regard, it is essential to analyse these variables in padel to understand their implications for performance, particularly in relation to playing position and match result.

Therefore, the aims of the present study were (1) to compare the physiological responses and neuromuscular capacity of amateur padel players during match play according to match outcome (Win or Loss) and playing position (Right Side [RS] vs. Left Side [LS]) and (2) to examine the progression of these physiological responses and neuromuscular fatigue across sets as a function of match outcome and playing position. We hypothesised that players who Win and those playing on the Left Side would show higher physiological demands and neuromuscular fatigue and that physiological responses would progressively increase throughout the match.

## 2. Materials and Methods

### 2.1. Participants

Fifty-two male amateur padel players (35.6 ± 11.6 years) participated in the present study, selected through non-probabilistic convenience sampling. Each player took part in a single match, resulting in a total of 13 matches analysed. The mean duration of the matches was 57.2 ± 15.7 min. Specifically, the mean duration of set one was 21.7 ± 6.2 min, set two lasted 20.9 ± 4.4 min, and set three, when played, lasted 28.2 ± 7.2 min. In total, matches comprised an average of 152.0 ± 40.4 points, with set one including 60.1 ± 15.3 points, set two 56.4 ± 9.4 points, and set three 72.6 ± 18.3 points. Players were grouped according to match outcome: the group of players who Won (Win, *n* = 26, 35.3 ± 11.3 years) and the group who Lost (Lose, *n* = 26, 36.1 ± 12.0 years). Additionally, players were categorised by playing position: 50% (*n* = 26, 36.81 ± 13.39 years) played on the Left Side (LS), while the remaining players (*n* = 26, 34.65 ± 9.52 years) played on the Right Side (RS). All participants met the pre-established inclusion criteria: a minimum of one year of regular padel experience, absence of injuries in the three months prior to the study, and no medical contraindications that could affect physical or motor performance. Importantly, all players were habitual padel practitioners, with varying levels of weekly training frequency: 25% (n = 13) trained 1–2 times per week, 40% (n = 21) trained 3 times per week, and 35% (n = 18) trained 4 or more times per week. The average duration of the matches was 57.2 ± 15.7 min. Specifically, the mean duration of set one was 21.7 ± 6.2 min, set two lasted 20.9 ± 4.4 min, and set three, when played, lasted 28.2 ± 7.2 min. The study adhered to the guidelines set out in the Declaration of Helsinki [16] and received approval from the Ethics Committee for Research Involving Human Participants at the University of the Basque Country (UPV/EHU) (CEISH M10/2024/167). 

### 2.2. Procedure

**Figure 1:**
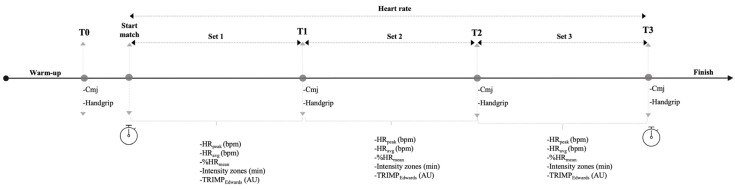
Experimental procedure of the study, evaluation phases, and monitoring during the padel match. Measurements conducted before the match (T0) and after each set (T1, T2, T3) are shown, including countermovement jump (CMJ) and maximal handgrip strength, along with the monitoring of physiological variables during competition. **Note:** HR_peak_ (bpm): peak heart rate reached in each set; HR_avg_ (bpm): average heart rate during each set; %HR_mean_: percentage of the average heart rate relative to the peak value; TRIMP_Edwards_ (AU): internal match load in arbitrary units.

The experimental sessions were conducted at a certified sports facility to ensure consistency in measurement procedures. Matches were organised according to the competitive level of the players, as determined by an internal ranking system used by the sports performance centre. This system adjusts players’ levels based on their previous match results, thereby ensuring balanced pairings. Importantly, all players participated from their usual and exclusive playing position (Right Side or Left Side), which corresponds to the side they consistently occupy during both training sessions and official competitions. Prior to the start of each match, all participants completed a general warm-up consisting of five minutes of continuous moderate-intensity running, followed by joint mobility exercises and dynamic drills designed to activate the major muscle groups involved in padel. The warm-up protocol for all players was designed, led, and supervised by the research team. Following the warm-up, baseline assessments (T0) were conducted, which included, in order, the countermovement jump (CMJ) test and the isometric handgrip strength test. After these initial assessments, the players participated in a padel match following the traditional doubles format, according to the official rules of the International Padel Federation [4] and with comparable playing levels among the four participants in each match. During the matches, the following variables were recorded for each player: heart rate, set and total match duration, results of each set and of the match, and the number of points played. Immediately after the conclusion of each set [T1 after the first set (S1), T2 after the second set (S2), and T3 after the third set (S3), if applicable], players repeated the CMJ test and the handgrip strength assessment (Figure 1).

### 2.3. Measurements

*Vertical jump capacity:* To assess vertical jump capacity, a countermovement jump (CMJ) was performed using both legs. During the jump, participants kept their hands on their hips in order to isolate the lower limbs’ ability to generate explosive force and eliminate the influence of arm movement [17]. In the countermovement phase, participants were allowed to choose the degree of hip and knee flexion that they found most comfortable and effective [18,19]. Flight height (cm) was measured using an optical data collection system (Opto Jump Next^®^, Microgate, Bolzano, Italy).

*Isometric forearm strength capacity:* Isometric forearm strength was assessed using the handgrip test. Participants performed the test with the arm extended downwards along the vertical axis [20]. The test was carried out using the dominant hand, defined as the one habitually used to hold the racket. Measurements were taken using a hand dynamometer (5030j1, Jamar^®^, Sammons Preston, Inc., Nottinghamshire, UK).

*Heart rate:* Heart rate (HR) was recorded continuously throughout each match using a portable monitor (Polar V800, Kempele, Finland), as in recent studies [21]. The collected data were transferred and analysed using Polar Flow software (version 4, Polar, Kempele, Finland). The following variables were obtained for each set and the entire match: (i) peak heart rate (HR_peak_), (ii) average heart rate (HR_avg_), and (iii) percentage of the average heart rate relative to the peak heart rate (%HR_mean_).

Additionally, the time spent in different intensity zones was analysed, based on the percentage of HR_peak_ and consistent with previous research [22,23]. These zones were defined as follows: low (Z1) (<75% HR_peak_), moderate (Z2) (75–85% HR_peak_), high (Z3) (85–95% HR_peak_), and maximal (Z4) (>95% HR_peak_). Internal load for each set or the full match (TRIMP_Edwards_) was also quantified [24]. To calculate this, the accumulated time (min) spent in each intensity zone was multiplied by a corresponding weighting factor: zone 5 (90–100% HR_peak)_ = 5 points, zone 4 (80–89% HR_peak_) = 4 points, zone 3 (70–79% HR_peak_) = 3 points, zone 2 (60–69% HR_peak_) = 2 points, and zone 1 (50–59% HR_peak_) = 1 point. The total internal load was then obtained by summing the resulting values from all zones [24,25,26].

### 2.4. Statistical Analysis

Results are presented as means ± standard deviations. Data were assessed for normality and homogeneity of variance using the Shapiro–Wilk and Levene’s tests, respectively, to determine the use of parametric or non-parametric tests as appropriate. To compare LS and RS players, as well as Win and Lose players, across various variables and time points, the independent samples *t*-test was applied when the assumptions of normality and homogeneity of variance were met. When either assumption was violated, the Mann–Whitney *U* test was used. To analyse within-group differences across match time points for both physiological responses (S1, S2, and S3) and test outcomes (T0, T1, T2, and T3), a repeated-measures ANOVA with Bonferroni post hoc tests was conducted. In addition, to simultaneously examine the effects of group (LS vs. RS or Win vs. Lose) and time point (S1, S2, S3 or T0, T1, T2, T3), a two-way repeated-measures ANCOVA (2 × 3 or 2 × 4) was performed, also with Bonferroni post hoc comparisons. Effect sizes for pairwise differences were calculated using Cohen’s *d* for parametric variables and the rank biserial correlation coefficient (*r_b_*) for non-parametric variables. The qualitative interpretation of effect sizes was as follows: *d* = < 0–0.1 (no effect), 0.2–0.4 (small effect), 0.5–0.7 (intermediate effect), and 0.8–≥ 1.0 (large effect) [27] and *r_b_* = < 0.10 (very small), 0.10–0.29 (small), 0.30–0.49 (moderate), and ≥ 0.50 (large) [28]. All statistical analyses were performed using JASP software (JASP for macOS, version 0.18.3, Amsterdam, The Netherlands). The level of statistical significance was set at *p* < 0.05.

## 3. Results

### 3.1. Physiological Responses by Match Outcome and Playing Position

Table 1 presents the results and differences in players’ physiological responses across the full match and each set, according to match outcome (Win vs. Lose) and playing position (RS vs. LS). No significant differences were observed between players who Won and those who Lost, nor between RS and LS players, in any of the physiological response variables analysed.

### 3.2. Physiological Evolution by Match Outcome

**Figure 2:**
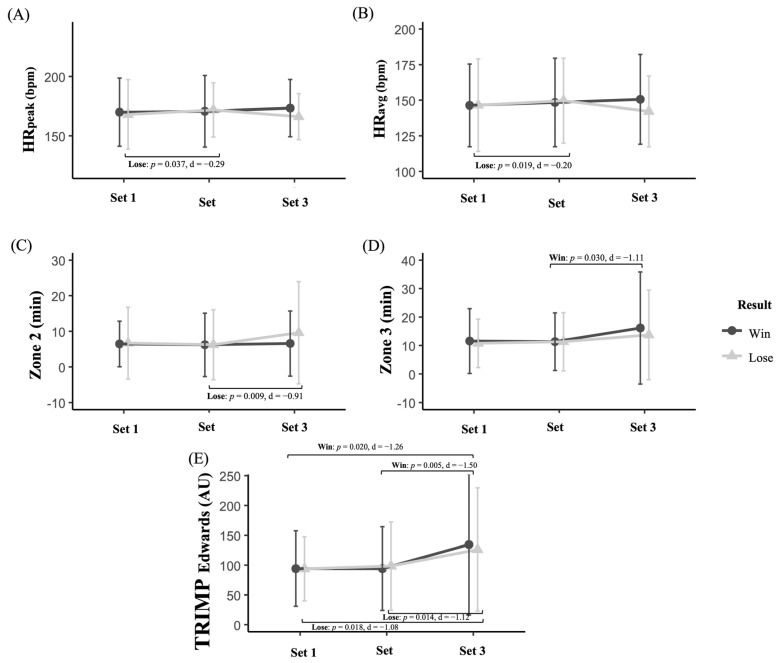
Evolution of physiological responses in amateur padel players according to match outcome (Win vs. Lose): (**A**) Peak heart rate (HR_peak_), (**B**) average heart rate (HR_avg_), (**C**) time in zone 2, (**D**) time in zone 3, and (**E**) TRIMP_Edwards_ across the different sets.

Regarding the evolution of physiological responses across sets during the match based on match outcome (Figure 2), players in the Win group showed a higher TRIMP_Edwards_ in S3 compared to S1 (*p* = 0.020, *d* = −1.26, large). Similarly, Win group players spent more time in Z3 (*p* = 0.030, *d* = −1.11, large) and had a higher TRIMP_Edwards_ (*p* = 0.005, *d* = −1.50, large) in S3 compared to S2. In contrast, players in the Lose group recorded higher HR_peak_ (*p* = 0.037, *d* = −0.29, small) and HR_avg_ (*p* = 0.019, *d* = −0.20, small) in S3 compared to S1. Similarly, players in the Lose group spent more time in Z3 (*p* = 0.009, *d* = −0.914, large) and showed a higher TRIMP_Edwards_ (*p* = 0.014, *d* = −1.12, large) in S3 than in S2. Finally, the repeated-measures ANCOVA analysis revealed no significant differences in any of the physiological variables in the interaction between match outcome (Win vs. Lose) and match stage (S1–S3).

### 3.3. Physiological Evolution by Playing Position

**Figure 3:**
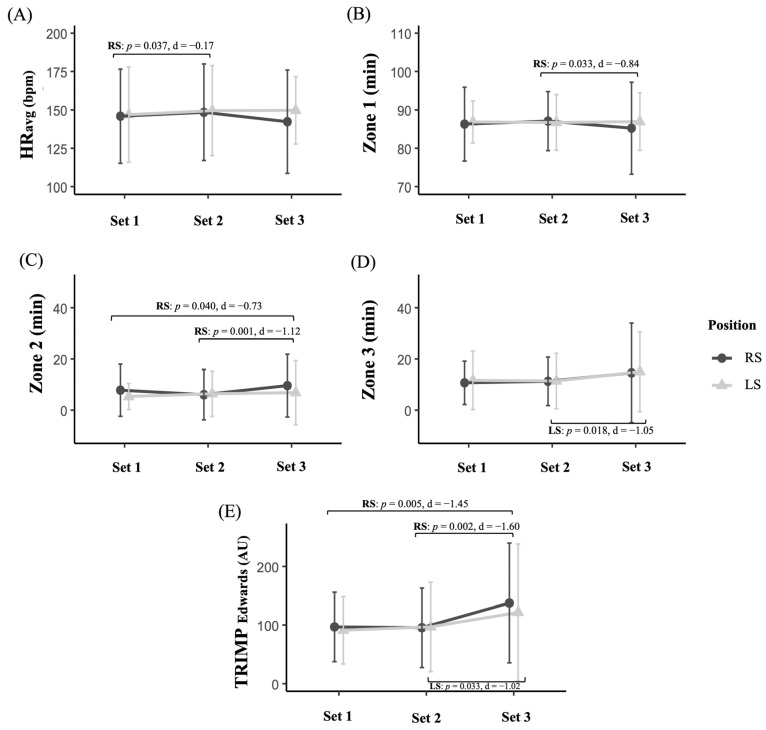
Evolution of physiological responses in amateur padel players according to playing position (Right Side [RS] vs. Left Side [LS]): (**A**) Peak heart rate (HR_avg_), (**B**) time in zone 1, (**C**) time in zone 2, (**D**) time in zone 3, and (**E**) TRIMP_Edwards_.

Regarding physiological responses throughout the match based on the on-court role, different patterns emerged depending on the playing position (Figure 3). Among players in the RS group, significant changes were identified in several variables. For HR_avg_, a significant increase was observed between S1 and S2 (*p* = 0.037, *d* = −0.17, no effect). Concerning time spent in different heart rate zones, significant increases were recorded in Z1 between S2 and S3 (*p* = 0.033, *d* = −0.84, large) and in Z2 between S1 and S3 (*p* = 0.040, *d* = −0.73, intermediate), as well as between S2 and S3 (*p* = 0.001, *d* = −1.12, large). Regarding TRIMP_Edwards_, RS players showed significant increases from S1 to S3 (*p* = 0.005, *d* = −1.45, large) and from S2 to S3 (*p* = 0.002, *d* = −1.60, large). Players in the LS group also showed significant changes. A significant increase in time spent in Z3 was observed between S2 and S3 (*p* = 0.018, *d* = −1.05, large). Similarly, TRIMP_Edwards_ significantly increased in S3 compared to S2 (*p* = 0.033, *d* = −1.02, large). Finally, the repeated-measures ANCOVA revealed no significant differences in any of the physiological variables analysed (HR_peak_, HR_avg_, %HR_mean_, Z1, Z2, Z3, Z4, and TRIMP_Edwards_) for the interaction between playing position (RS vs. LS) and match stage (S1–S3).

### 3.4. Neuromuscular Responses by Match Outcome and Playing Position

Table 2 presents the results for jump capacity and handgrip strength at the different time points (T0–T3) for players in the Win and Lose groups, as well as for RS and LS players. Significant differences were observed only concerning playing position in the handgrip test at T1 and T2, with LS players displaying significantly higher values. However, no significant differences were found between players who Won or Lost at any point during the match.

### 3.5. Neuromuscular Evolution by Match Outcome and Playing Position

**Figure 4:**
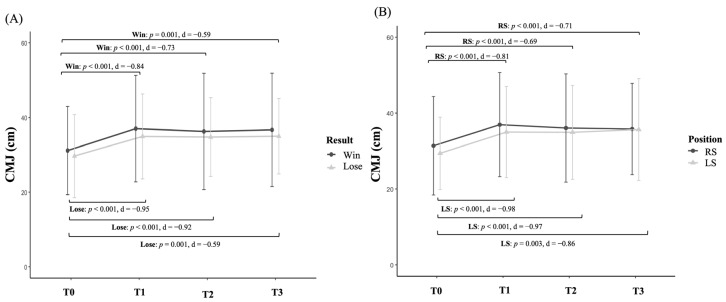
Evolution of countermovement jump (CMJ) performance in amateur padel players according to match outcome (**A**) and playing position (**B**).

Regarding the evolution of neuromuscular responses across sets during the match based on match outcome (Figure 4A), players in the Win group showed significant increases in CMJ between T0 and T1 (*p* < 0.001, d = −0.84, large), T0 and T2 (*p* < 0.001, d = −0.73, intermediate), and T0 and T3 (*p* = 0.001, d = −0.59, intermediate). Similarly, players in the Lose group showed significant increases in CMJ between T0 and T1 (*p* < 0.001, d = −0.95, large), T0 and T2 (*p* < 0.001, d = −0.92, large), and T0 and T3 (*p* < 0.001, d = −1.11, large). When examining the evolution of neuromuscular responses throughout the match based on playing position (Figure 4B), RS players exhibited significant increases in CMJ between T0 and T1 (*p* < 0.001, d = −0.81, large), T0 and T2 (*p* < 0.001, d = −0.69, intermediate), and T0 and T3 (*p* < 0.001, d = −0.71, intermediate). Similarly, LS players also showed significant improvements in CMJ between T0 and T1 (*p* < 0.001, d = −0.98, large), T0 and T2 (*p* < 0.001, d = −0.97, large), and T0 and T3 (*p* = 0.003, d = −0.86, large).

No significant changes were observed in handgrip strength throughout the match, regardless of match outcome or playing position. Lastly, the repeated-measures ANCOVA revealed no significant interactions between match outcome (Win vs. Lose) or playing position (RS vs. LS) and match stage (T0–T3) in any of the neuromuscular variables analysed.

## 4. Discussion

Previous studies have described heart rate as a valuable tool for quantifying and monitoring effort during physical activity [29,30]. However, the available evidence on physiological responses and their progression throughout padel matches, particularly among amateur players, remains limited. Therefore, the aims of the present study were (1) to compare the physiological responses and neuromuscular capacity of amateur padel players during match play according to match outcome (Win or Loss) and playing position (Right Side [RS] vs. Left Side [LS]) and (2) to examine the progression of these physiological responses and neuromuscular fatigue across sets as a function of match outcome and playing position. The findings of the present study indicate that no significant differences were observed in physiological load between players positioned on the RS and LS of the court nor between those who Won or Lost the match, both overall and across the individual sets. Nevertheless, significant within-group variations were identified throughout the match, suggesting a progressive increase in physiological responses as play continued. In contrast, no evidence of neuromuscular fatigue was found in any of the variables analysed, regardless of match outcome or playing position (RS vs. LS).

### 4.1. Differences in Physiological Responses Based on Playing Position and Match Outcome

Although previous research in padel has examined physiological responses during competition [21,31], few studies have explored whether these responses differ according to match outcome or playing position. Understanding whether physiological differences exist between winners and losers, as well as between court positions, could offer valuable insights for optimising the physical and strategic preparation of padel players. The findings of the present study did not reveal any significant differences in the physiological responses analysed during the competition (HR_peak_, HR_avg_, %HR_mean_, Z1–Z4, and TRIMP_Edwards_) between players according to either playing position or match outcome in any of the sets or across the match as a whole. These findings suggest that cardiovascular demand is similar regardless of courtside or match outcome. These results contrast with those reported by Roldán-Márquez et al. [30] in individual tennis players, who showed higher HR_avg_ and HR_peak_ values, as well as differences in time distribution across intensity zones, among those who Lost compared to those who Won. The discrepancies between studies may be attributed to the distinct characteristics of padel and tennis, such as court dimensions and match format (doubles versus singles). Although tennis and padel share certain similarities, tennis, particularly in the singles format, imposes greater physical and cardiovascular demands than padel [32]. This could explain why [30] observed differences in cardiovascular responses based on match outcome in tennis, whereas the present study did not detect such differences in padel. In individual tennis, losing a match may involve adopting a more reactive role, responding to the opponent’s dominant strokes and covering greater distances, which results in spending more time in high-intensity zones [30]. In contrast, in padel, the smaller court dimensions and the tactical use of walls may account for the lack of significant increases in physiological demands when a player or team is behind in the score.

### 4.2. Evolution of Physiological Responses Across Different Sets of the Match

The evolution of physiological responses across different sets in padel has been scarcely addressed in the literature, particularly when considering variables such as match outcome or playing position. The analysis of different match moments, according to match outcome and position, revealed significant differences. Regarding the match outcome, players who Won showed significant increases in TRIMP_Edwards_ and time spent in Z3 as the match progressed. Players who Lost also exhibited increases in heart rate (HR_peak_ and HR_avg_) between S1 and S3, as well as in TRIMP_Edwards_ and time spent in zone 2 during the third set. In a similar vein, Villafaina et al. [8] examined heart rate variation at three time points (pre-match, during the match, and post-match) in padel players. They found no significant differences between the winners and losers. However, within each group (winners and losers), significant differences were observed in variables such as HR_avg_, R-R interval time, and stress index. In this regard, Bustamante-Sánchez et al. [15] reported that sympathetic nervous system activation intensifies in the final sets of a match, as these moments are decisive and require greater physiological effort. Therefore, padel players should incorporate high-intensity game situations into their training, characterised by elevated heart rate responses, in order to promote prolonged exposure to specific conditions of competitive stress. One of the novel contributions of the present study is that the evolution across sets was not the same for winners and losers. Among players who Lost, significant increases were observed throughout the match in HR variables (HR_peak_ and HR_avg_) between S1 and S2, time spent in zone 2 between S2 and S3, and TRIMP_Edwards_ between S1 and S3 and between S2 and S3. In contrast, players who Won showed significant increases in time spent in zone 3 between S2 and S3, and in TRIMP_Edwards_ between S1 and S3 and between S2 and S3. These results suggest that, although no between-group differences were found in physiological responses, each group exhibited a distinct progression in their responses throughout the match. Furthermore, in relation to playing position, RS players showed a significant increase in HR_avg_ from S1 to S3. Significant increases were also observed among RS players between S1 and S3 in Z2 and TRIMP_Edwards_, as well as between S2 and S3 in Z1, Z2, and TRIMP_Edwards_. Additionally, LS players showed significant increases only between S2 and S3 in Z3 and TRIMP_Edwards_. In contrast to the present study, Parraca et al. [21] reported no significant differences in heart rate variability across the three time points evaluated during the match (30, 60, and 90 min of play) in amateur padel players. These discrepancies may be explained by the fact that, in the current study, the time points were defined by specific sets and further segmented by playing position and match outcome. This level of detail allowed for the detection of variations that may go unnoticed in more generalised analyses. These differences in the evolution of physiological responses between groups may be attributed to tactical decisions made during the match. At certain moments, play may become concentrated on one side of the court or focus on a specific player, thereby influencing the physical load experienced and, consequently, the physiological response. Given that physiological responses in padel have been understudied, particularly in relation to playing position and match outcome, these findings are especially relevant for understanding how such responses evolve throughout a match. This knowledge can contribute to improving training planning, promoting specificity and adaptation based on each player’s individual needs and contextual match factors such as scoreline or court position.

### 4.3. Neuromuscular Fatigue According to Playing Position and Match Outcome

Quantifying potential fatigue in padel may offer valuable insights for optimising performance, planning recovery and training strategies more effectively, and contributing to injury prevention. In this regard, regardless of match outcome or playing position, all subgroups improved their jump performance throughout the match, showing statistically significant differences between T0 and the subsequent time points between sets (T1–T3). Specifically, the improvement in CMJ performance observed across all groups may be attributed to the post-activation performance enhancement (PAPE) effect. This phenomenon occurs when a prior intense activity induces neuromuscular potentiation, resulting in improved performance in explosive exercises, such as the CMJ [33,34]. According to Fischer and Paternoster [33], PAPE can be explained by several physiological mechanisms, including increased muscle temperature, heightened neuromuscular excitability, and improved efficiency in actin–myosin cross-bridge coupling. Moreover, Sun et al. [34] found that the effectiveness of PAPE depends on the balance between neuromuscular potentiation and fatigue, emphasising that when the recovery time between the activation activity and the subsequent test is appropriate, CMJ performance is optimised and residual fatigue is minimised. This may explain the findings observed in the padel players in the present study, where pauses between points and breaks between sets may have allowed for partial recovery, preventing excessive fatigue accumulation and promoting the expression of the PAPE effect. Unlike sports involving continuous and sustained efforts [35] or even intermittent efforts with short recovery periods [36], the intermittent nature of padel may help preserve or even enhance jump performance throughout the match.

Regarding handgrip strength, significant differences were observed at T1 and T2. LS players demonstrated higher levels of handgrip strength compared to RS players at both T1 and T2. This positional difference may be explained by the profile of LS players, who typically adopt a more offensive role and execute a greater number of smashes and powerful strokes, which may require and lead to the development of greater grip strength. In this respect, Ramón-Llin et al. [37] reported that players positioned on the LS perform significantly more cross-court and winning shots than those on the RS. However, no significant within-group differences were found when comparing the different time points (T0, T1, T2, and T3). This suggests that no fatigue occurred in handgrip strength during the match, regardless of whether the players Won or Lost or whether they played on the RS or LS. The absence of differences across sets may indicate that, despite the duration of the match, the neuromuscular load associated with gameplay was not sufficiently demanding to induce fatigue in handgrip strength. It would be advisable for future research to analyse fatigue in other key muscle groups, such as the shoulder musculature, given its involvement in repetitive striking and overhead actions.

Despite the methodological rigour employed, this study presents certain limitations that should be considered when interpreting the results. First, the sample consisted of amateur padel players selected through non-probabilistic convenience sampling, which may limit the generalizability of the findings to other playing levels, such as professional or novice players. Future studies could expand the sample and include players from different competitive levels to assess potential differences in physiological and neuromuscular responses. Second, although several variables were controlled during the study, external factors such as fatigue status, sleep quality, nutrition, and other pre-competition conditions that could influence performance were not recorded or analysed. These variables may affect physiological performance levels and should be taken into account in future research. Third, it is important to consider that playing style can vary considerably between players, even within the same competitive level. This variability may influence in-game physical demands and fatigue responses and represents a potential confounding factor when interpreting the results. Despite these limitations, the findings provide valuable insights into the physiological responses of amateur padel players, laying the groundwork for future studies exploring the effects of competitive load and fatigue mechanisms in this sport.

## 5. Conclusions

The present study analysed the evolution of physiological responses and neuromuscular fatigue in amateur padel players according to playing position and match outcome. The findings indicate that no significant differences in physiological load were observed between RS and LS players or between winners and losers, either across the individual sets or in the match as a whole. However, changes were identified within each group throughout the matches, showing a progressive increase in physiological responses as the sets progressed. On the other hand, no signs of neuromuscular fatigue were detected using the selected assessment methods in any of the groups. The improvement in CMJ performance in the first set and its subsequent stabilisation may be related to the post-activation performance enhancement (PAPE) effect, facilitated by the intermittent nature of padel, which allows for partial recovery periods between efforts. These results provide relevant evidence on physiological and neuromuscular responses in padel and suggest that amateur padel training should include high-intensity scenarios to address increasing physiological demands. Improved neuromuscular performance supports tailored activation strategies, and positional differences in grip strength highlight the need for role-specific training.

## Figures and Tables

**Table 1:** Physiological response results according to match outcome (Win or Lose) and playing position (Left Side [LS] and Right Side [RS]) for the entire match and across the different sets.

		HR_peak_ (bpm)	HR_avg_ (bpm)	%HR_mean_	Z1 (min)	Z2 (min)	Z3 (min)	Z4 (min)	TRIMP_Edwards_ (AU)
**Match outcome**									
Whole match	Win (n = 26)	173.00 ± 14.28	147.42 ± 15.46	85.11 ± 3.74	6.12 ± 7.48	18.28 ± 7.43	29.69 ± 13.32	5.42 ± 5.74	242.29 ± 74.87
	Lose (n = 26)	174.69 ± 10.64	147.71 ± 14.51	84.41 ± 4.08	8.53 ± 7.96	21.05 ± 11.56	28.13 ± 12.15	4.37 ± 3.14	249.93 ± 64.26
	ES Win vs. Lose	0.02	−0.13	0.18	−0.21	−0.28	0.07	0.06	−0.07
Set 1 (S1)	Win (n = 26)	169.92 ± 14.37	146.35 ± 14.54	86.09 ± 3.69	1.44 ± 1.54	6.44 ± 3.20	11.57 ± 5.70	2.54 ± 1.80	94.13 ± 31.60
	Lose (n = 26)	168.00 ± 14.66	146.46 ± 16.25	87.07 ± 4.09	2.00 ± 2.60	6.67 ± 5.04	10.76 ± 4.26	2.87 ± 1.59	93.82 ± 26.87
	ES Win vs. Lose	−0,01	−0.07	−0.14	−0.07	0.08	0.07	−0.18	0.19
Set 2 (S2)	Win (n = 26)	170.65 ± 15.10	148.36 ± 15.52	86.85 ± 3.13	1.59 ± 2.93	6.19 ± 4.45	11.36 ± 5.06	2.94 ± 2.04	94.09 ± 35.08
	Lose (n = 26)	171.81 ± 11.41	149.63 ± 14.90	86.96 ± 4.27	2.00 ± 2.41	6.22 ± 4.91	11.34 ± 5.11	3.59 ± 3.00	98.35 ± 36.91
	ES Win vs. Lose	−0.16	−0.07	−0.03	−0.14	−0.08	−0.03	−0.06	−0.041
Set 3 (S3)	Win (n = 13)	173.33 ± 12.06	150.56 ± 15.76	86.77 ± 5.29	2.75 ± 3.89	6.56 ± 4.57	16.16 ± 9.84	5.78 ± 6.59	134.56 ± 59.17
	Lose (n = 13)	166.14 ± 9.69	142.13 ± 12.45	85.50 ± 4.81	3.85 ± 5.41	9.60 ± 7.18	13.71 ± 7.86	3.61 ± 2.99	126.14 ± 51.63
	ES Win vs. Lose	0.66	0.60	0.53	−0.03	−0.49	0.28	0.19	0.39
**Playing position**									
Whole match	RS (n = 26)	172± 11.75	146.51 ± 15.59	84.55 ± 4.55	8.85 ± 9.81	21.68 ± 11.05	28.61 ± 13.39	5.53 ± 6.03	257.09 ± 66.61
	LS (n = 26)	174.73 ± 13.38	148.62 ± 14.29	84.97 ± 3.17	5.81 ± 4.60	17.64 ± 7.89	29.21 ± 12.12	4.26 ± 2.49	235.13 ± 71.26
	ES RS vs. LS	−0.18	−0.10	−0.11	0.03	0.42	−0.04	−0.02	0.18
Set 1 (S1)	RS (n = 26)	168.88 ± 12.86	145.90 ± 15.32	86.29 ± 4.81	2.02 ± 2.64	7.78 ± 5.10	10.69 ± 4.23	2.71 ± 1.91	96.81 ± 29.67
	LS (n = 26)	169.04 ± 16.06	146.92 ± 15.50	86.86 ± 2.74	1.42 ± 1.39	5.32 ± 2.55	11.65 ± 5.71	2.69 ± 1.48	91.15 ± 28.70
	ES RS vs. LS	−0.13	−0.01	−0.15	0.06	−0.06	−0.04	−0.19	0.28
Set 2 (S2)	RS (n = 26)	170.19 ± 13.16	148.46 ± 15.74	87.08 ± 3.85	1.89 ± 3.32	6.03 ± 4.93	11.28 ± 4.74	3.24 ± 2.51	95.48 ± 33.88
	LS (n = 26)	172.27 ± 13.54	149.53 ± 14.68	86.73 ± 3.63	1.71 ± 1.85	6.38 ± 4.43	11.41 ± 5.41	3.29 ± 2.66	96.96 ± 38.12
	ES RS vs. LS	−0.13	−0.07	0.10	−0.15	−0.01	0.01	−0.02	−0.12
Set 3 (S3)	RS (n = 13)	166.69 ± 11.65	142.30 ± 16.81	85.23 ± 5.99	4.62 ± 6.04	9.58 ± 6.14	14.57 ± 9.74	4.82 ± 6.05	137.63 ± 51.05
	LS (n = 13)	172.23 ± 10.51	149.74 ± 11.00	86.95 ± 3.76	2.01 ± 2.31	6.81 ± 6.26	15.04 ± 7.82	4.31 ± 3.62	121.54 ± 58.23
	ES RS vs. LS	−0.30	−0.52	−0.34	−0.23	−0.49	0.28	−0.11	0.39

Note: HRpeak (bpm): peak heart rate reached in each set; HRavg (bpm): average heart rate during each set; %HRmean: percentage of the average heart rate relative to the peak value; Z1 = heart rate zone 1 (<75% of HRpeak); Z2 = heart rate zone 2 (75–85% of HRpeak); Z3 = heart rate zone 3 (85–95% of HRpeak); Z4 = heart rate zone 4 (>95% of HRpeak); TRIMPEdwards: internal match load in arbitrary units. Italics = parametric.

**Table 2:** Results for jump performance and handgrip strength at different time points during the match (T0, T1, T2, and T3) for the Win and Lose groups and for players in the Right Side (RS) and Left Side (LS) positions.

		T0	T1	T2	T3
**Match outcome (Win or Lose)**					
CMJ	Win	31.13 ± 5.91	37.02 ± 7.13	36.26 ± 7.79	36.68 ± 7.59
	Lose	29.67 ± 5.57	34.93 ± 5.68	34.76 ± 5.28	34.99 ± 5.06
	ES Win vs. Lose	0.26	0.33	0.23	0.27
Handgrip	Win	55.2 ± 8.7	53.5 ± 9.1	53.7 ± 10.2	51.8 ± 8.6
	Lose	52.3 ± 9.5	49.1 ± 9.3	50.0 ± 10.8	51.0
	ES Win vs. Lose	0.32	0.48	0.35	0.08
**Playing position (RS or LS)**					
CMJ	RS	31.40 ± 6.49	36.95 ± 6.86	36.08 ± 7.13	35.81 ± 6.03
	LS	29.40 ± 4.78	35.00 ± 6.02	34.94 ± 6.19	35.66 ± 6.71
	ES RS vs. LS	0.35	0.30	0.17	0.02
Handgrip	RS	52.11 ± 7.48	48.10 ± 7.51	48.85 ± 10.14	50.36 ± 10.22
	LS	55.35 ± 10.47	54.51 ± 10.03	54.88 ± 10.30	52.47 ± 8.10
	ES RS vs. LS	−0.36	−0.72 *	−0.59 *	−0.23

Note: T0: baseline assessment; T1: after set 1; T2: after set 2; T3: after set 3; RS: Right Side; LS: Left Side; * *p* < 0.05.

## Data Availability

The datasets generated and/or analysed during the present study are not publicly available due to confidentiality agreements with the participants.

## Abbreviations

The following abbreviations are used in this manuscript:

%HR_mean_	Percentage of the Average Heart Rate Relative to the Peak Heart Rate
AU	Arbitrary Units
CMJ	Countermovement Jump
ES	Effect Size
HR_avg_	Average Heart Rate
HR_peak_	Peak Heart Rate
LS	Left Side
RS	Right Side
S1	Set 1
S2	Set 2
S3	Set 3
T0	Pre-Match Time Point
T1	Post-Set 1 Time Point
T2	Post-Set 2 Time Point
T3	Post-Set 3 Time Point
TRIMP_Edwards_	Training Impulse by Edwards
Z1	Heart Rate Zone 1 (<75% HRpeak)
Z2	Heart Rate Zone 2 (75–85% HRpeak)
Z3	Heart Rate Zone 3 (85–95% HRpeak)
Z4	Heart Rate Zone 4 (>95% HRpeak)
